# Abnormal basic visual processing functions in binocular fusion disorders

**DOI:** 10.1038/s41598-023-46291-w

**Published:** 2023-11-07

**Authors:** Laura Benhaim-Sitbon, Maria Lev, Uri Polat

**Affiliations:** School of Optometry and Vision Sciences, Faculty of Life Sciences, Bar-Ilan University, Ramat Gan, Israel

**Keywords:** Sensory processing, Neuroscience, Visual system

## Abstract

Heterophoria is a common type of binocular fusion disorder that consists of a latent eye misalignment with potential consequences on daily activities such as reading or working on a computer (with CVS). Crowding, a type of contextual modulation, can also impair reading. Our recent studies found an abnormal pattern of low-level visual processing with larger perceptive fields (PF) in heterophoria. The PF is the fundamental processing unit of human vision and both masking and crowding depend on its size. We investigated how heterophoria would impact the PF’s size via a lateral masking experiment and consequently affect the foveal crowding at different letter-spacings (the crowding zone). More specifically, we explored the relationship between crowding, lateral masking, the PF’s size, and the amount of heterophoria. The binocular horizontal PF’s size was larger with heterophoric subjects, in agreement with our previous study. We found a stronger crowding and an extended crowding zone associated with slower response times; this shows that the processing of letter identification under both crowded and uncrowded conditions requires more processing effort in heterophoric individuals. In agreement with previous studies, we found a correlation between the crowding zone and the PF’s size; each was strongly correlated with the amount of phoria. These findings resemble those involving the PF size and the extended crowding found at the fovea in amblyopia and young children. We suggest that these findings could help explain the inter-observers’ variability found in the masking literature, and the reading difficulties often encountered in subjects with high heterophoria.

## Introduction

According to recent surveys, an average person spends about 7 h daily on electronic devices, accounting for 38–43% of their waking hours^1^. Professionals spend approximately 5 h per day on work-related and personal email^2,3^, while college students dedicate 15 h weekly to academic tasks^4,5^. Medical students may study on average, 10.6 h a day for exams^6^. These activities put significant demands on the quality of binocular fusion.

Although we have two eyes, we usually perceive one single image under normal viewing conditions. Binocular fusion is one perceptual outcome of binocular vision processing through which information from both eyes is combined, enabling single vision (rather than double vision). Binocular fusion relies on sensory fusion (matching visual inputs in size, sharpness, and brightness) and motor fusion, achieved through vergence eye movements, to align the two images globally.

Heterophoria is a prevalent sensorimotor disorder (up to 35.6% in the adult population^7–9^). It consists of a latent misalignment of the eyes, which becomes apparent when binocular fusion is disrupted, usually by occluding one eye. Note that this misalignment is not manifest like in strabismus but remains concealed until an artificial interference with the sensory binocular inputs is introduced (such as occlusion of one eye). Routine examinations of binocular vision by specialists^10,11^ include assessing the type (horizontal, vertical, or cyclorotary) and the amount of misalignment. Horizontal phoria is the most common type, where the eyes converge (esophoria) or diverge (exophoria)^9^. The amount of misalignment can vary with the distance. The distribution of heterophoria within the general population is significantly non-normal, and a high incidence of approximate orthophoria (indicating proper eye alignment without latent misalignment) has been observed, particularly at a distance^12^. This orthophorization results from successful fusion adaptation enabled by tonic vergence and normal physical or mechanical factors. However, if these factors deviate from the normal limits or if the fusion adaptation process fails to develop adequately, a significant heterophoria may arise^13^.

Although not always pathological (see *Supplementary Material*—“Table 1: Physiological ranges for heterophoria in the literature”, 10.1038/s41598-022-16458-y), clinically significant heterophoria can lead to symptoms and visual discomfort, particularly in computer users. Computer Vision Syndrome (CVS) is a prevalent condition affecting up to 75% of computer users^14–16^, causing issues like headaches, fatigue, and blurry vision. The computer vision syndrome (CVS) is a group of eye-related conditions and symptoms such as headaches, fatigue, or blurry vision, which appear as a result of prolonged use of display devices^17^. Individuals with heterophoria may experience reduced endurance and increased visual discomfort when working on computers^18^. Heterophoria can impact daily visual activities, including reading^19^. Efficient binocular vision enables a unified perception of text, leading to benefits such as reduced fixation times and increased reading speed. Recent research has revealed that individuals with heterophoria experience a diminished binocular advantage in terms of fixation times and reading speed, and that the degree of this disadvantage correlates with the severity of their phoria^20^. Recently, it was suggested that subjects with binocular fusion disorders, such as horizontal heterophoria, exhibit an abnormal pattern of low-level visual processing^21,22^.

Visual crowding, the difficulty in recognizing objects when surrounded by other objects, can contribute to poor reading performance. Excessive crowding is linked to slow reading^23^, as it limits the size of the uncrowded visual span, which is the range of letters that can be reliably recognized without moving the eyes^24^. The critical spacing for letter identification, the smallest distance between letters to avoid crowding, is predictive of both critical spacing and reading span^24^. Reading speed is more influenced by letter spacing than by letter size^25^. Crowding is commonly associated with slow reading in dyslexia^26–28^ and is prevalent in the amblyopic population^29,30^. Dyslexic individuals have difficulties decoding words despite adequate instruction, intelligence, and intact sensory abilities (for a review, see^31,32^).

Visual crowding is a type of contextual modulation that affects various aspects of vision, including reading^23^, Vernier acuity^33,34^, orientation discrimination^35,36^, face recognition^37,38^, moving stimuli^39,40^, and real-world scenes^41^. It impairs object perception in both peripheral^42–45^and central vision^45–48^, as well as in amblyopic^30,49–51^ and developing vision^52,53^. The phenomenon is explained by different theories at various stages in the visual hierarchy^54,55^, ranging from low-level receptive fields to high-level attention. For instance, the Attentional Resolution theory, which involves top down effects^56^, proposes that cueing can reduce crowding^57,58^, whereas the Configural Grouping theory suggests crowding arises when target and flankers are similar. Crowding occurs when the target and the flankers overlap within the same neural unit^47,59–61^, analogously to visual masking^47^.

The fundamental unit of low-level visual information analysis is the receptive field (RF)^62^. It is a specific region of the sensory space where a stimulus can modify the firing of a neuron^63^. RF is influenced by lateral interactions in the primary visual cortex (V1)^64–66^, which involve stimulation (facilitation) or inhibition (suppression) by neighboring neurons via long-range connections between similar orientation columns. Visual masking experiments reveal these lateral interactions, modulating visual response sharpness and contrast^67–69^. In such experiments, the perception of a target like a Gabor Patch can be enhanced (facilitation) or diminished (suppression) based on the distance from collinear flankers. Note that in the literature, lateral masking refers to situations in which the target and the flankers are presented concomitantly or with a delay. This delay may indicate that the mask appears before or after the target stimulus (see Polat and Sagi ^70,71^). The RF has a psychophysical counterpart termed the perceptive field (PF), estimated to range from 2λ to 3λ at the fovea^70,72–74^ and about 5λ at an eccentricity of 4°^61,75–77^. Lev and Polat^61^ suggested and showed^47,78^ that the distance at which suppression turns to facilitation in the LM experiments is indicative of the PF's size. The crowding zone refers to the spatial area surrounding a target where the presence of flankers inhibits target identification, typically around 3–5 arcmin for central vision for moderate to infinite presentation times^60,79^, but they can be greater than 6 arcmin with shorter presentation times^46,47^. Note that in our study the term “suppression” refers specifically to the psychophysical phenomenon observed in visual masking experiments, where the perception of a target is diminished. This differs from interocular suppression, which prevents double vision by suppressing one image.

Lateral interactions are believed to play a role in contextual modulation, affecting both crowding and masking^61^. These phenomena are linked at both the fovea^47,53,70,80^ and the periphery^61^, relying on the perceptive field (PF) size and on spatio-temporal parameters such as presentation times, spatial frequency, and eccentricity^47^. There is an ongoing debate whether they share certain spatio-temporal characteristics or are distinct phenomena (Lev & Polat^47^). Notably, the tasks involved in these phenomena differ: masking involves contrast detection, whereas crowding involves object orientation identification.

Despite their common dependence on PF, assessing the PF size is primarily carried out using a lateral masking paradigm rather than crowding. This method has gained credibility in recent studies^47,78^ as a reliable approach for evaluating the PF size, correlating well with crowding effects across central and peripheral vision^47^. Although a similar assessment using crowding is conceivable, it poses greater challenges, since crowding involves complex interactions that make assessing the PF’s size less straightforward compared with the lateral masking paradigm. Specifically, PFs transmit their processing of features to integration fields^81–84^ that are involved in visual crowding^84,85^. Integration fields are larger areas that gather information from multiple receptive fields; they facilitate the integration of features and contextual information from neighboring regions, aiding in tasks such as object recognition, scene understanding, and spatial relationships^81–84^. The development of the visual system shapes the crowding zone and the PF's size^52,53^. Notably, the PF’s size and crowding can also be modified by perceptual learning ^23,86–90^. Amblyopic subjects tend to have larger PF sizes compared to neurotypical subjects, regardless of the type of amblyopia (anisometropic or strabismic) ^91,92^.

Recently, Benhaim-Sitbon et al.^21^ found abnormal lateral interactions only for the horizontal meridian in individuals with high horizontal heterophoria. Another study^22^ showed larger binocular perceptive field size along the horizontal meridian in subjects with high horizontal heterophoria, resembling findings in meridional amblyopia^91,92^. However, a link between these results and crowding in high heterophoric subjects required further investigation. Given that crowding and masking both depend on perceptive field size^78^, we hypothesized that high heterophoria could impact crowding due to latent eye misalignment and its effect on binocular fusion. If so, since some studies suggest that crowding sets a limit on reading speed^24,50,85,93^, we think that this could corroborate the difficulties in binocular reading recently found in the high heterophoric population^20^. This research aimed to assess the crowding effect for different letter spacings in the foveal region, associated response times, crowding zone, and perceptive field size for the horizontal meridian in a high heterophoric population.

Importantly, we found a significantly larger crowding effect for all letter-spacings as well as significantly slower response times associated with the high heterophoria group. The crowding zone was significantly larger and was correlated with the amount of heterophoria. In agreement with our previous findings, we found that the PF’s sizes were greater for the phoric group and were correlated with the amount of heterophoria.

## Methods

### Subjects

Thirteen subjects, from 19 to 36 years old (27.46 ± 6.09, mean ± STD) participated in the 2 experiments in the study. Each subject was included after a comprehensive orthoptic examination by a certified orthoptist. The orthoptic assessments, which included both sensory and motor assessments, were the same as in a previous study^21^ (see *Supplementary Material*: 10.1038/s41598-022-16458-y for details), except for the stereoacuity, which was measured by the Randot Stereotest (Stereo Optical, Inc., Chicago, IL) and the angle of phoria, measured by the alternating cover test (ACT) only. Each subject had normal or corrected-to-normal visual acuity (both monocular and binocular), normal stereoscopic vision (a minimum of 25 arcsec), and fusion at all viewing distances (assessed with Bagolini striated glasses). In accordance with our previous studies^21,22^, we classified individuals into the heterophoria group based on the presence of a horizontal phoria equal to or greater than 6Δ (prism diopters) and/or a vertical phoria equal to or greater than 2Δ at least at one of the testing distances (distances that were intermediate at one meter or nearer). In both groups the subjects did not exhibit any clinical signs of accommodative disorders, amblyopia, stereopsis disorders, strabismic problems, any decompensation to intermittent strabismus, or ocular disease (exclusion criteria for both groups).

The study protocol was approved by the Internal Review Board (IRB) of Bar-Ilan University. Informed consent was obtained from all subjects and/or their legal guardian(s). All methods were performed in accordance with the relevant guidelines and regulations.

### Apparatus and stimuli

For both types of experiments, we used an in-house-developed software for psychophysical experiments (PSY) developed by Bonneh^94^.

### Crowding experiment

We used a Microsoft PC tablet with a 23 cm screen. The screen resolution was set at 1920 × 1280 pixels. The size of a single pixel was 0.12 cm. For a viewing distance of one meter, it represented a 13° × 8.5° area. Thus, 1° includes around 148 pixels, and 1 arcminute (arcmin) includes approximately 2.5 pixels (1920/13), which correspond to the standard vision of 6/6 (20/20). The stimuli consisted of a letter E presented either alone or within a matrix of other E letters. The size of the letter E was 15 pixels (or 5 arcmin); thus, the size of each gap between the E strokes, which determines the acuity limit, was 3 pixels (the letter E is composed of 5 gaps). Since the acuity limit is determined by the resolution of the gaps between the E strokes, and since 3 pixels correspond approximately to 1 arcmin, the letter E corresponded approximately to an acuity of 6/6 (20/20).

### Lateral masking experiment

We presented the stimuli on a BENQ XL 2411 color monitor connected to a PC controlled by a NVDIA GTX 710 video card, using an in-house-developed software for psychophysical experiments (PSY) developed by Y.S. Bonneh^94^. The monitor resolution was 1920 × 1080 pixels, and gamma correction was applied.

Stimuli were localized gray-level gratings (Gabor patches) with equal wavelength (λ) and standard deviation (STD, σ), which allowed a minimum of 2 cycles by Gabor patch (GP). Each GP had a frequency of 8 cycles per degree (cpd, λ = 0.125°). Thus, each GP measured 15 arcmin (2λ*60). The GPs were modulated from a background luminance of 40 cd/m^2^.

### Procedures

The results of our previous study suggested that the perceptive field is extended in the heterophoric population^22^. Since both crowding and masking are dependent on the PF’s size and since crowding is like masking in certain spatial and temporal conditions, we decided to investigate the behavior of the heterophoric population via two different sets of experiments: One is a crowding experiment to determine whether the heterophoria population exhibits a larger crowding effect and a broader crowding zone; the other is a lateral masking experiment to assess the perceptive field. Note that crowding and masking both depend on the perceptive field (PF) size. The masking experiment allows for a direct assessment of the PF’s size, whereas crowding involves a more complex mechanism that makes assessing the PF’s size less straightforward. Both experiments took place at one meter sitting distance with binocularly viewing.

### Crowding experiment

**Figure 1:**
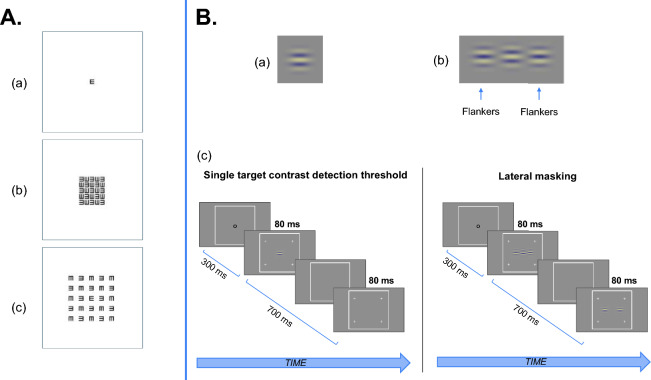
(**A**) Illustration of some presentations during the crowding experiment. (**a**) A single letter E, (**b**) a crowded condition with 0.25 inter-letter spacing, and (**c**) a crowded condition with 1.5 inter-letter spacing. (**B**) Illustrations of the lateral masking experiment: (**a**) a GP with an orientation of 0°, (**b**) a target GP and two flankers with a target-flanker separation of 3λ, (**c**) an illustration of the lateral masking paradigm. Contrast was enhanced for illustration purposes.

The aim of this experiment was to determine whether the heterophoric population displayed a stronger crowding effect and whether the crowding effect persisted for larger letter spacing in comparison with a control population. The task and procedure were similar to previous studies in our lab^47,95^. The targets were black tumbling E patterns that appeared on a white background at the center of the screen with a duration of 40ms (see Fig. 1A). A forced-choice paradigm was used: the subjects were asked to choose the direction of the central target E (right or left) by pressing the right or the left mouse key. The uncrowded condition (a single letter E) and the different crowded conditions were presented randomly; each presentation was indicated by four peripheral high-contrast crosses. The crowded conditions consisted of a matrix of E letters arranged randomly around the E target letter. The size of the matrix was 5 × 5 letters. We used 6 different letter spacings between the letters (0.25, 0.5, 0.75, 1, 1.25, and 1.5 letter spacing). There were 80 trials per condition, which represented a total of 560 trials per experiment (7 conditions × 80). Each experiment was repeated three times for each subject. The performance of each subject was recorded as the percentage of correct responses (the percent correct, %). The crowding effect was calculated as the difference between the percentage of correct responses of the single letter condition and the crowded condition (the percent correct for the single letter minus the percent correct for the crowded condition). Auditory feedback was given for each incorrect response. Practice trials were utilized to familiarize participants with the task. Response times were measured as the time from the stimulus onset to a response^46,95–98^.

### Lateral masking experiment

Since it was suggested in previous studies that there was an abnormal pattern of lateral interations^21^ and an extended perceptive field^22^ only in the horizontal meridian for subjects presenting a high horizontal heterophoria, and since both masking and crowding are dependent on the perceptual field size^47,52,91,99^, we wanted to compare the PF’s size between the two groups and to assess the correlation between the crowding zone and the PF obtained with lateral masking.

The experiment was similar to our standard contrast detection in our lab^21,47,70^. We used Gabor patches with a global orientation of 0 degree (see Fig. 1B) to maximize the potential effect on the PF size in the heterophoric group, since it was previously found that the PF’s size is extended for the horizontal meridian^22^. Six different target-to-flanker separations (λ) were used: 1.5, 2, 2.5, 3, 3.5, and 4 λ (see Fig. 1B). Two conditions were tested (see Fig. 1B): a contrast detection threshold of a single Gabor Patch (sGP) without and with the presence of two collinear GP flankers having a contrast of 60% (LM). To measure the central GP target contrast threshold, we used a two-alternative temporal forced-choice paradigm and a 3:1 staircase procedure (converging to 79% correct responses)^100^. We also used flankers with an opposite phase to maximize the effect, since we observed in our previous study that the difference in the PF’s size obtained between the phoria and control group with opposite phase flankers was greater than with an equal phase^22^.

The subjects had to report which of the two stimulus presentations contained the target GP by pressing a mouse button (left for the first one and right for the second one). Auditory feedback was given for incorrect responses. Four peripheral high-contrast crosses indicated the GP presentations (stimulus intervals). At the beginning of each trial, the subjects were instructed to maintain their fixation at the center of a screen, which was denoted by a small circle. Blinking was permitted but the subjects had to avoid eye movements during the trials. When ready, they pressed the middle button of the mouse to activate a trial sequence: a no-stimulus interval of 300 ms with a temporal jitter of 500 ms (0–500 ms with equal distribution) and two sequential stimulus presentations (80 ms each) that were separated by another no-stimulus interval (700 ms + temporal jitter up to 500 ms). The stimulus intervals were randomly ordered and only one of the two contained the target; for the LM condition, both contained the mask (see Fig. 1B).

The GP amplitude and the distance between the target and flankers were kept constant for each trial. Throughout the experiment, a peripheral lock, which consisted of a square sustaining 15.4° visual degrees horizontally and vertically from the center, was used to limit phoria decompensation during the absence of stimuli (and/or the interstimulus interval). Screen luminance remained the same during the stimulus and the no-stimulus intervals. The experiment was repeated three times for each subject. Practice trials were utilized to familiarize participants with the task.

### Crowding zone and PF estimation

#### Crowding experiment

**Figure 2:**
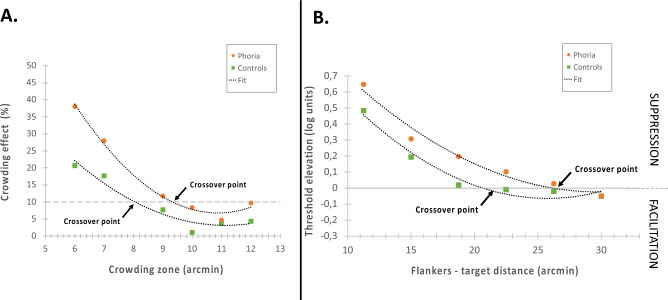
These illustrations represent an estimation of the crowding zone (**A**) and the PF size (**B**) for one control subject and one heterophoric subject. (**A**) Crowding effect (%) as a function of the crowding zone (in arcmin). The circles filled in orange denote the results obtained for one phoric subject (S1). The squares filled in green denote the results obtained for one control subject (S9). The fitted line is denoted by a black dotted line. The gray dashed line denotes the limit of a 10% crowding effect. For each subject separately, the data was plotted, and the crowding zone was estimated by the crossover point between the fitted line and the 10% crowding effect. (**B**) Threshold elevation (in log units) as a function of the flanker-target distance (in arcmin). The orange circles denote the results obtained for one phoric subject (S6). The green squares denote the results obtained for one control subject (S12). The fitted line is denoted by a black dotted line. The data were plotted for each subject separately, and the PF size was estimated by identifying the specific point where the fitted line intersected with the null threshold elevation (y = 0) axis, indicating the transition from suppression to facilitation.

The crowding zone refers to the spatial area surrounding a target within which the presence of flankers (here the E matrix) impedes target identification. To determine the crowding zone, we first estimated the crowding for each subject by calculating the crowding effect (the percentage of correct responses for the E target minus the percentage of correct responses for the crowded condition) for each crowding effect as a function of the letter spacing (calculated in arcmin). Then, we fitted a polynomial line for which the equation was $y = ax^{2} - bx + c$. Since we observed a floor effect around a crowding effect of 10% for the heterophoric group, we decided to set this limit to assess the crowding zone for each group. The crowding zone corresponded to the crossover point between the fitted line and the 10% crowding effect (see Fig. 2A).

#### Lateral masking experiment

It has been suggested^47,61,70,73^ and found in previous studies^47,78^ with a lateral masking experiment that the distance at which the suppression turns to facilitation provides an estimate of the size of the PF. Although assessing the perceptive field (PF) size using crowding is possible, it is more challenging due to the intricate mechanisms involved, making assessing the PF’s size less straightforward than with the lateral masking paradigm. For each subject, we fitted a polynomial line for which the equation was $y=a{x}^{2}-bx+c$, and we used the crossover point where collinear suppression was transformed to facilitation (y = 0) as the crossing border of the PF^47,52,61^ (see Fig. 2B).

### Data and statistical analysis

Repetitions for each condition were averaged for each subject and all data points were confirmed as not being outliers. We calculated the average median time for each subject^97^.

We used a two-way mixed ANOVA to test the effect of 2 nominal variables (such as group and letter spacing) on a continuous outcome (the crowding effect). We graphically checked the normality of the residual homogeneity of variance assumptions. We performed some linear mixed effect models, and the ANOVA was conducted on the resulting models. Subject ID was defined as a random effect and nominal variables as fixed effects. All interactions were included in the initial models; however, the models were refitted without non-significant interactions. The post-hoc analyses were completed as pairwise comparisons defined by linear contrasts. To control for multiple testing, the false discovery rate (FDR) was corrected. Whenever the interactions were removed, we performed the post-hoc analysis by averaging the non-interacting factors.

A Welch two-sample t-test with unequal variance was performed to test the effect on one nominal variable (group) for a continuous outcome (contrast detection thresholds, the crowding zone, the response time, or the PF’s size).

Pearson’s and Spearman’s correlation tests were conducted between the perceptive field sizes obtained with the lateral masking experiment and the crowding experiment, and between the PF size obtained with the crowding experiment and the angle of the phoria.

## Results

### Crowding experiment

#### Subjects

Thirteen subjects participated in the experiment as either the heterophoric group or the control group (see Table 1). The two groups were not statistically different in terms of age or stereoacuity but differed in their phoria (for statistical details, see below). Seven subjects, with an average age of 28.14 ± 6.47 years old (mean ± SD), were assigned to the heterophoric group and six subjects with an average age of 26.66 ± 6.47 years old (mean ± SD) were included in the control group (a mean difference of 1.48 years old, t(0.4204), *p* = 0.6838). At one meter, the average amount of phoria for the heterophoric group was 6.57 ± 1.21 Δ (mean ± SE) and for the control group it was 1.00 ± 0.68Δ (t(-3.817), *p* = 0.0029). The maximum amount of phoria was measured at 40cm where the heterophoric group exhibited an average phoria of 11.57 ± 1.54 Δ (mean ± SE) and the control group exhibited an average phoria of 1.83 ± 0.74 Δ (mean ± SE) (t(-5.3774), *p* < 0.001). Both the heterophoric and control groups had a similar stereoacuity (group: mean ± SE; heterophoria: 21.42 ± 0.92 arcsec; control: 20.83 ± 0.83 arcsec, t(0.4719), *p* = 0.6462). No subjects reported double vision or intermittent double vision during the experiment.

**Table 1:** Clinical orthoptic details and participation of the enrolled subjects in the experiments. F female, M male, ortho orthophoria, X exophoria, E esophoria, X' exophoria at a near distance, E' esophoria at a near distance, NPC Near Point of convergence, Δ prismatic diopter, cm centimeters.

Subjects	Gender	Age	Groups	Cover test			Stereoacuity (arcsec)	Step convergence (break) in Δ		NPC (cm)	Experiment(s)
				4 m	1 m	40 cm		4 m	40 cm		
S1	F	36	Phoria	E12	E12	E′10	20	40	40	3	All
S2	F	32	Phoria	X4	X6	X′14	20	16	20	7	All
S3	M	35	Phoria	X4	X6	X′7	20	12	18	7	All
S4	F	23	Phoria	X2	X4	X′6	20	8	12	8	Crowding
S5	F	21	Phoria	X2	X4	X16	25	12	18	6	Crowding
S6	F	23	Phoria	X2	X12	X16	20	14	20	6	All
S7	F	27	Phoria	X2	X4	X12	25	16	20	7	All
S8	F	23	Control	Ortho	Ortho	Ortho	20	18	25	4	All
S9	F	33	Control	Ortho	Ortho	X2	20	20	25	4	All
S10	F	19	Control	Ortho	Ortho	Ortho	20	18	20	6	All
S11	M	36	Control	E2	E2	E2	20	20	30	4	All
S12	M	25	Control	Ortho	Ortho	X2	20	18	35	9	All
S13	F	24	Control	Ortho	X2	X4	25	4	6	15	Crowding

#### Crowding effect

**Figure 3:**
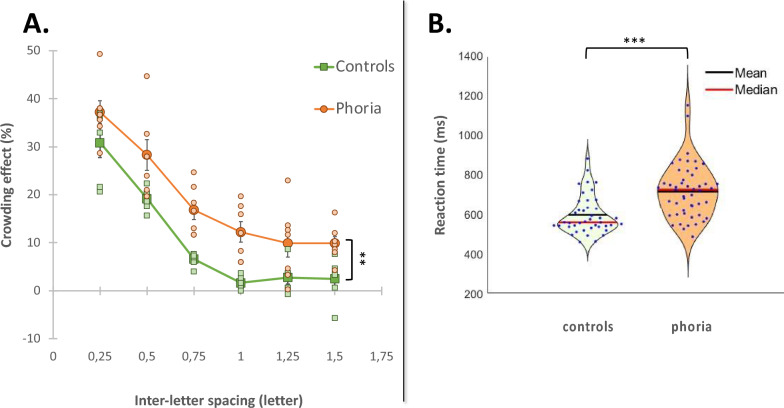
(**A**) Average crowding effect (%) for the heterophoric group (n = 7) versus the control group (n = 6) as a function of the inter-letter spacing (in letters). The heterophoric group is denoted by a filled orange circle and the control group by a filled green square. Additionally, smaller light orange circles and small light green squares denote the crowding effect for individual subjects in the heterophoria and control groups, respectively. The error bar denotes the standard deviation of the mean. (**B**) The violin plots represent the distribution of the response times (ms) during the crowding experiment for the control group (n = 6), denoted in light green, and for the phoric group (n = 7) in dark orange. Each violin plot contains the results of both the crowded and the uncrowded conditions. ***p ≤ 0.001, **p ≤ 0.01, *p ≤ 0.05.

We wanted to determine whether the heterophoric group exhibited a larger crowding effect than the control group. Briefly, we measured the crowding effect (percent of correct responses for the single E minus the percent of correct responses for the crowded condition) for each inter-letter spacing. The average results per group are presented in Fig. 3. We found that for all inter-letter spacings, the heterophoric group presented a larger crowding effect than the control group (*p* = 0.0016). We performed a two-way ANOVA to test the effect of group and inter-letter spacing on the crowding effect. Group (F (1,11) = 17.3746, *p* = 0.0016) and the inter-letter spacing (F (5,55) = 88.3238, *p* < 0.0001) had a constant effect on the crowding, although they did not depend on each other (F (5,55) = 0.498, *p* = 0.7763): this is well illustrated in Fig. 3A, where we can observe that the difference between the control and phoria is constant.

#### Response time

We then compared the average median of the two groups’ response times obtained for all letter spacings; the results are presented in Fig. 3B. We found that the heterophoria group exhibited globally longer response times than the control group for the crowding experiment (group: mean ± SE; heterophoria: 713.71 ± 19.91 ms; controls 593.35 ± 15.02 ms, t(− 4.8888), *p* < 0.0001). Note that the phoria group's data exhibit two instances of notably high response times (RT). It is important to emphasize that these high RT points do not qualify as outliers. Nevertheless, we conducted a thorough analysis to ascertain whether these specific data points contribute significantly to the observed differences. The results showed that even after removing the two highest RT points, the differences observed within the phoria group remain statistically significant (*p* = 0.0000229 with all the data, and *p* = 0.0000487 without the two most extreme points). The heterophoria group’s response times were longer for both the uncrowded condition (group: mean ± SE; heterophoria: 637.28 ± 42.09 ms; controls 517.16 ± 26.35 ms, t(− 2.4188), *p* = 0.0366) and for the crowded conditions (group: mean ± SE; heterophoria: 726.45 ± 21.70 ms; controls 606.05 ± 16.34 ms, t(− 4.5238), *p* < 0.0001). The results are in agreement with previous studies^47,95^.

#### Crowding zone

We plotted the crowding effect as a function of the inter-letter spacing (in arcmin) for each subject; the crowding zone was assessed for each subject (see the “Crowding zone and PF estimation”). We found that the heterophoric group had a larger crowding zone than the controls (group: mean ± SE; heterophoria: 10.67 ± 0.55 arcmin; controls 8.30 ± 0.06 arcmin, t(− 4.2933), *p* = 0.0049).

#### Correlation with phoria

**Figure 4:**
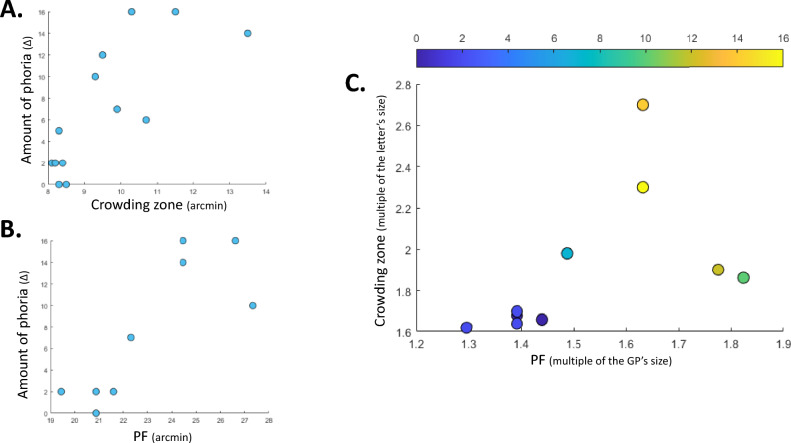
(**A**) Amount of phoria (in Δ) as a function of the crowding zone (arcmin). (**B**) Amount of phoria (in Δ) as a function of the PF’s size (arcmin). (**C**) Crowding zone (a multiple of the letter’s size) as a function of the PF’s size (a multiple of the GP’s size); the color bar denotes the amount of phoria (in Δ).

To determine whether the crowding zone was correlated with the amount of phoria, we performed both Pearson’s and Spearman’s correlation tests. We found a moderately high correlation for both (Pearson’s rho = 0.7703, Spearman’s rho = 0.7781) and both were significant (*p* = 0.0021 and *p* = 0.0017, respectively). The results are illustrated in Fig. 4A.

### Lateral masking experiment

#### Subjects

A total of ten subjects that participated in the crowding experiment (see Table 1) also participated in the lateral masking experiment (5 heterophoric subjects and 5 controls). The groups differed in terms of phoria but were not statistically different in terms of age and stereoacuity (see below for the statistical details). The heterophoric group was 30.6 ± 5.50 years old (mean ± SD) and the control group was 27.2 ± 7.08 years old (mean ± SD); the difference in the average was not significant (t(0.8474), *p* = 0.4214). At one meter, the amount of phoria measured was 7.6 ± 7.08Δ (mean ± SE) for the heterophoric group and 0.4 ± 7.08Δ (mean ± SE) for the control group (t(− 6.9338), *p* < 0.001). A maximum angle of phoria was measured at 40 cm (group: mean ± SE; heterophoria: 11.8 ± 1.56Δ, controls: 1.2 ± 0.49Δ, t(− 4.7270), *p* = 0.0015). The stereoacuity was under 25 arcsec for the two groups (group: mean ± SE, heterophoria: 21 ± 1 arcsec, controls 20 ± 0 arcsec, t(1), *p* = 0.3466). There was no report of intermittent double vision during the experiment.

#### PF assessment

First, the contrast detection thresholds for single targets were not statistically different between the two groups (group: mean ± SE; heterophoria: 0.84 ± 0.04 log unit; controls 0.74 ± 0.02 log unit, t(− 2.1235), *p* = 0.0755).

We plotted the threshold elevation (the contrast for the single target minus the contrast for the lateral masking condition) for each target-flankers’ separation converted to arcmin, and we assessed the PF (see “Crowding zone and PF estimation”). We found that the PF’s size was significantly higher for the heterophoric group than for the controls (group: mean ± SE; heterophoria: 25.06 ± 1.23 arcmin; controls 20.736 ± 0.35 log unit, t(− 4.4972), *p* = 0.0058). These results are in agreement with our previous study^21^.

#### Correlation with phoria

We also found a high significant correlation between the amount of phoria and the PF’s size (Pearson’s rho = 0.8099, *p* = 0.0045; Spearman’s rho = 0.7875, *p* = 0.0068). The results are illustrated in Fig. 4B.

### Correlation between the crowding zone and the PF

We first normalized the crowding zone to the size of one letter (the crowding zone in arcmin/the size of the letter in arc min) and the PF’s size to the size of a GP (the PF’s size in arcmin/size of the GP in arcmin). Results are summarized on Fig. 4C. We found a moderately high significant correlation with the Spearman’s test (rho = 0.7655, *p* = 0.0099). With the Pearson’s test, we found a moderate correlation, but it was not significant (rho = 0.5162, *p* = 0.1267).

## Discussion

Binocular fusion is one perceptual outcome of binocular vision processing that enables single vision through which information from the two eyes is combined to enable single vision. Heterophoria is a common type of binocular fusion disorder that consists of a latent eye misalignment with potential consequences for daily activities such as reading or working on a computer (with CVS) when the misalignment is not well compensated by the vergence system (high magnitude of the phoria or poor fusional reserves) or the orthophorization process. Crowding, a type of contextual modulation, can also impair reading. It refers to the difficulty in recognizing an object when it is presented in clutter. Visual masking, another type of contextual modulation that reveals the lateral interactions, refers to the modified perception of a target when surrounded by flankers. Like visual masking, crowding is thought to be mediated by lateral interactions in V1^61^.

Since we recently showed that subjects who present high heterophoria were characterized by (a) an abnormal pattern and an asymmetry of the lateral interactions^21^, (b) an extended and asymmetric PF size^22^, and since crowding and masking (which may be similarly affected by the lateral interactions^61^) are both dependent on the PF’s size under specific spatio-temporal parameters^47^, we hypothesized that subjects with high heterophoria would exhibit a larger crowding effect and a larger crowding zone. We investigated how the high heterophoria would impact the foveal crowding for short presentation times and at different letter-spacings, as well as the crowding zone, the PF’s size via a lateral masking experiment, and explored the relationship between crowding, lateral masking, the PF’s size, and the amount of heterophoria. Importantly, we found that the high heterophoric subjects presented a stronger crowding and an extended crowding zone. These results were associated with slower response times than for the controls, showing that the processing of letter recognition under both crowded and uncrowded conditions required more processing effort. The binocular horizontal PF’s sizes were also larger with high heterophoric subjects, in accordance with our previous study^22^. In agreement with previous results^22^, we found a correlation between the crowding zone and the PF’s size, and each was strongly correlated with the amount of phoria. These findings resemble those of the PF size and the extended crowding found at the fovea for populations with visual developmental disorders such as amblyopia but without being attributed to abnormal refraction or manifest strabismus. In addition, we suggest that our findings could contribute to explaining the high inter-subject variability found in a previous study^47^.

### A larger strength and extent of crowding: a pseudo-amblyopic behavior

Across all letter-spacings, the heterophoric group showed a significantly larger crowding effect. We observed a crowding effect from 20 to 734% larger for the heterophoric group, depending on the inter-letter spacing. At shorter inter-letter spacings, the difference was less, since the control group also exhibited a large crowding effect, in agreement with the findings of the literature^47^. However, this difference was pronounced at spacing where usually the crowding effect disappears in normal findings^47^: at one letter spacing the crowding effect was 7.5 times larger for the heterophoric group than for the control group. This difference decreased but persisted at larger inter-letter spacings where the crowding strength was 3.5 times higher than that of the controls. These results resemble the larger foveal crowding that was observed in strabismic amblyopia^51,59,85^ but without being attributed to manifest strabismus. Note that amblyopia results from a lack of normal maturation of the visual system, which leads to reduced visual acuity^49,101,102^, diminished contrast sensitivity^101,103,104^, and especially at high spatial frequencies^81,101,103^, impaired spatial interactions ^92^ and a slower reading speed. Recently, it was shown that the crowding strength and extent are larger in the immature visual system of infants and that only with normal development will the crowding decrease to reach its standard effect at 5–6 years old^52,53^. Thus, we suggest that these findings could point out that high heterophoria behaves like a developmental disorder.

Within the domain of binocular fusion disorders and ocular alignment, we observed a wide spectrum of eye misalignment. At one end of this spectrum lies heterophoria, which signifies the natural inclination of the eyes to deviate from perfect alignment without active binocular fusion. Notably, higher levels of heterophoria can predispose individuals to more pronounced fixation disparities and sometimes to microstrabismus. To gain a deeper understanding of the association between high heterophoria and the sensory phenomena observed in our study (larger PF and an enhanced crowding effect), in the future we will explore the impact of fixation disparities, microstrabismus, and interocular suppression.

1. Fixation disparities, positioned in the middle of this spectrum, signify alignment discrepancies during active fixation. In our previous study^21^, where we monitored eye fixation during a lateral masking experiment with an eye tracker device, no discernible differences in eye fixation or manifest misalignment were observed between the control group and the heterophoria group. Strikingly, the heterophoria group displayed suppression, whereas the control group exhibited facilitation. This finding suggests that fixation disparities are unlikely to be the causal factor behind the sensory effects observed.
2. Microstrabismus occupies an intermediate position along this spectrum between fixation disparities and manifest strabismus, indicating subtle misalignments detectable through specialized clinical assessments. It is worth noting that, as detailed in the “Methods” section, all participants in our study exhibited 20/20 visual acuity in each eye, excellent stereoscopic vision (approximately 21 arcsec for the heterophoria group), and fusion without central scotomas when tested with Bagolini lenses. Consequently, these findings do not support the presence of microstrabismus in our subjects^105^.
3. Interocular suppression can emerge as a compensatory mechanism to maintain single vision and alleviate discomfort. The persistence of interocular suppression over time can have adverse implications for visual development, analogous to the relationship between strabismus and amblyopia. Nevertheless, as previously discussed, it is unlikely that fixation disparity is the driving force behind this phenomenon, and clinical evidence does not support the presence of microstrabismus in our population. Consequently, the theory of interocular suppression explaining the sensory effects in our subjects is rendered less likely.

It is important to acknowledge that the eye tracking monitoring experiment^21^ was conducted with a relatively small sample size (five participants in the high heterophoria group). In our previous study^22^, we suggested that binocular instability from latent ocular misalignment^110^ and limited fusional reserves^111^ could explain the larger perceptive field (PF). Additionally, our previous study^21^ revealed an abnormal pattern of monocular lateral interactions in individuals with high heterophoria.

Given these observations, further investigations are warranted. These investigations should encompass assessing binocular instability, fixation disparities using standard clinical tests, and the monocular crowding effect in order to obtain a more comprehensive understanding of the relationship between high heterophoria and the sensory features observed in this study, specifically the larger PF and enhanced crowding effect.

In addition, we suggest that these results could explain the large inter-subject variability in the crowding effect observed by Lev and Polat^47^.

### Heterophoria requires an even more increased processing demand for crowded and uncrowded conditions

For both groups, we found that the response times for the crowded conditions were slower than for the uncrowded condition (around 15% slower for both groups). This result is in accordance with previous studies^46,95^ and indicates that extra processing time is required to overcome the effect of crowding in the fovea for stimuli with short presentation times. In a previous study, Lev and Polat^47^ suggested that the visual processing of stimuli presented briefly, which may induce a strong suppression, increases the processing efforts to rebalance the neural activity.

In general, the response times for the heterophoria group were found to be significantly longer than those of the control group, showing approximately a 20% slowdown (p < 0.0001, as shown in the response times). Specifically, when considering the uncrowded condition, the response times of the heterophoria group were approximately 23% slower than those of the control group. Moreover, under crowded conditions, the response times of the heterophoria group were approximately 20% slower than those of the control group. These results showed that (a) the processing of letter recognition under crowded conditions requires more processing effort, as revealed by the longer response time, i.e., the time needed for making decisions and (b) the processing effort is greater for the heterophoric group and persists for the uncrowded condition. It was suggested that the binocular combination of monocular inputs increases the processing load^95,106^ and since heterophoria is a binocular fusion disorder, we suggest that high heterophoria might add a processing demand on the binocular processing by adding more neural noise. Knowing that crowding produces a high processing load on the binocular visual processing, and that under ambiguous stimulation the neural noise within the visual system determines the responses^98^, we suggest that this could explain the even slower RT for the heterophoric group. These results are in agreement with the empirical speed-accuracy tradeoff function^95,107^. Considering the above, it could be interesting to further evaluate the crowding effect and determine whether the response times of the heterophoric group during monocular viewing would decrease to resemble the ones of the controls.

### Relationship between the PF and the crowding zone

The high heterophoria subjects presented a 20% larger PF size for the horizontal meridian than that of the controls, in agreement with our findings in a previous study^22^. Both the crowding zone and the PF’s size were correlated with the amount of phoria: the larger the phoria was, the larger the crowding and the PF’s size were. Since the size of GP (15 arcmin) was three times bigger than the size of the letter (5 arcmin), we decided to investigate the relationship between the PF and the crowding, once it was normalized by the size of their target. We observed that the crowding zone (expressed in multiples of letters), ranged from about 1.6 to 2.7, whereas the PF’s size ranged from about 1.3 to 1.8 (in multiples of the GP). Proportionally, the crowding zone was, on average, almost 25% larger than the PF’s size. One explanation for this difference could lie in distinguishing between the visual processing tasks for crowding and lateral masking. Crowding’s processing is divided into two processing stages^108^: an ‘early’ detection stage whereby only locations of high contrast in the image are selected, followed by an identification stage where the image intensity is used to identify the target (letter). In contrast, the visual masking processing implies only one detection stage. Thus, at short presentation times in the fovea, there is not enough time to overcome the crowding effect as previously shown^47^. In addition, we expected that the visual masking measured with an intermediate spatial frequency would have a medium size; however, the effect of crowding on letters may be determined by higher spatial frequencies. We suggest that this could explain the difference observed between the PF’s size and the crowding zone in our study. In addition, a similar difference was observed in the central field of strabismic amblyopes, where the strength and extent of crowding was greater than the ones for masking^109^. Considering that a pseudo-amblyopic behavior has been observed in high heterophoric populations^21,22^, we suggest that this could contribute to the difference between the crowding and masking extent observed in the high heterophoria population.

Given the findings from our previous studies indicating that high horizontal heterophoria exclusively affects lateral interactions and the PF’s size along the horizontal meridian^21,22^, coupled with the additional insights on crowding gained from the present study, we suggest that vertical phoria could similarly influence these factors along the vertical meridian. This necessitates further investigation to comprehensively understand the impact of vertical phoria on visual processing.

In our previous study^22^, we proposed that individuals with high heterophoria might have larger perceptive field (PF) sizes due to difficulties in maintaining their PFs overlaid, most likely caused by binocular instability from latent ocular misalignment^110^ and limited fusional reserves^111^. Given that crowding is influenced by the PF’s size, we suggest that the observed larger crowding zone in the heterophoria group could result from this phenomenon. To gain more insights, further investigations into monocular crowding and fixation disparities are warranted.

## Conclusion

High heterophoria affects the crowding strength and extent in the fovea similarly to amblyopia but without being attributed to abnormal refraction or manifest strabismus. In agreement with our recent study^22^, we found that high heterophoria affects the binocular PF’s size for the horizontal meridian. We suggest that these results could account for the large inter-subject differences found in Lev and Polat’s^52^ study and in other lateral masking literature^112,113^.

In agreement with previous studies^47,95^ and with the empirical tradeoff speed-accuracy function^107^, we found that the crowded conditions required a larger processing effort. Interestingly, we found that subjects with high heterophoria needed an even much greater processing effort. We believe that this additional processing demand could be attributed to the binocular combination system, which might be noisier in high heterophoria, considering its impact on binocular fusion.

Some studies^24,50,93^ suggest that crowding sets a limit on reading speed^85^. Considering our findings that the crowding strength and extent are much larger in high heterophoria and that crowding is positively correlated with the amount of phoria, we suggest that this could corroborate the difficulties in binocular reading recently found in the high heterophoric population, which were also correlated with the amount of phoria^20^.

## Data Availability

The datasets used and/or analyzed during the current study are available from the corresponding author on reasonable request.
